# Insecticides and testicular health: mechanisms of injury and protective natural products

**DOI:** 10.1007/s00210-025-04016-y

**Published:** 2025-03-26

**Authors:** Samar F. Darwish, Yasser M. Moustafa, Sherif S. Abdel Mageed, Ghaneya S. Hassan, Safwat Abdelhady Mangoura, Shaza H. Aly, Mai A. Mansour, Ahmed Amr Raouf, Al-Aliaa M. Sallam, Sylvia F. Fawzi, Asmaa M. Atta, Ola Elazazy, Walaa A. El-Dakroury, Aya A. El-Demerdash, El-Zahra M. Esmat, Mahmoud A. Elrebehy, Ahmed S. Doghish

**Affiliations:** 1 Pharmacology and Toxicology Department, Faculty of Pharmacy, Badr University in Cairo (BUC), Badr City, 11829 Cairo Egypt; 2 Pharmaceutical Chemistry Department, Faculty of Pharmacy, Badr University in Cairo (BUC), Badr City, 11829 Cairo Egypt; 3 Department of Pharmacognosy, Faculty of Pharmacy, Badr University in Cairo, Cairo, 11829 Egypt; 4 Department of Biochemistry, Faculty of Pharmacy, Badr University in Cairo (BUC), Badr City, 11829 Cairo Egypt; 5 Department of Pharmaceutics and Industrial Pharmacy, Faculty of Pharmacy, Badr University in Cairo (BUC), Badr City, 11829 Cairo Egypt; 6 Department of Clinical Pharmacy and Pharmacy Practice, Faculty of Pharmacy, Badr University in Cairo (BUC), Badr City, 11829 Cairo Egypt; 7 Department of Biochemistry, Faculty of Pharmacy, Galala University, New Galala City, 43713 Suez Egypt; 8 Biochemistry and Molecular Biology Department, Faculty of Pharmacy (Boys), Al-Azhar University, Nasr City, 11231 Cairo Egypt

**Keywords:** Insecticides, Testicular function, Potential hazards, Endocrine disruption, Natural products

## Abstract

In agriculture and public health, insecticides are vital chemicals that help manage diseases and control pests. However, their extensive use has raised concerns about their negative consequences on both humans and animals. Pesticide exposure impacts numerous human organs, including the reproductive system. Infertility is caused by reproductive system disorders, which is why they have received a lot of attention in recent decades. According to what is currently known, insecticides are among the substances that may lower the quality of the semen produced by exposed workers. The mechanisms of this action are still unclear, even though numerous underlying mechanisms have been suggested. With an emphasis on the harmful effects of insecticides on male reproductive processes, this review provides a thorough analysis of the toxicity profile of these substances. To reduce insecticides’ negative impacts on human and animal health and to direct future research initiatives, it is essential to comprehend their harmful consequences.

## Introduction

Crop yields meant for human consumption are at serious risk of declining as a result of pest encounters. According to studies, pest exposure results in the loss of more than half the yield of crops. As a result, controlling pests have helped raise crop productivity worldwide for a number of years (Uwamahoro et al. 2024). There are many different pesticides on the market that are used to target different kinds of pests, such as pyrethroids, organophosphates, phenylpyrazole, benzoylphenylurea, methylenedioxyphenyl, and organochlorines, and their usage is growing worldwide (Sabzevari and Hofman 2022).

Due to the widespread use of pesticides in both agriculture and healthcare, people and animals are exposed to pesticides either directly or indirectly throughout their daily activities (Mehrpour et al. 2014). Notably, even though pesticides are made to target particular creatures, there is still a significant chance that they will inadvertently harm organisms that are not their intended target (Rizzati et al. 2016). The most common ways that people are exposed to a combination of pesticides are through food, water, air, and tainted milk (Sharma et al. 2020). Human health hazards from pesticide exposure include headaches, nausea, and irritation of the skin and eyes (Lamichhane et al. 2019). Moreover, acute and/or chronic poisoning can arise from purposeful and inadvertent interaction with pesticides. They have negative effects on reproduction, which may result in infertility, in addition to their effects on the liver, kidneys, and heart (Hassan et al. 2021). As a result, a large number of pesticides that impact sexual differentiation and fertility are regularly and concurrently administered to humans and animals as endocrine disruptors (McKinlay et al. 2008). By decreasing sperm capacitation, sperm motility, motion parameters, and sperm cell viability, they lead to male infertility (Bae and Kwon 2021, Ali Abd El‐Rahman and Omar 2022).

In agriculture, organophosphate insecticides (OPs) are widely utilized. In mammals, OP, an ester of pentavalent phosphorus, shows a variety of toxicities (Abd-Elhakim et al. 2021). The irreversible phosphorylation of acetylcholinesterase (AchE) is the mechanism by which OP chemicals cause toxicity. As a result, acetylcholine levels rise, which activates nicotinic and muscarinic receptors and causes damage (Čolović et al. 2015). Many detrimental effects on many organs and blood variables, including immunotoxicity, carcinogenesis, endocrine development, reproductive toxicity, and delayed polyneuropathy, are brought on by the toxicity of OP pesticides (Pannu et al. 2021).

To fulfill our aim, the terms “insecticides,” “pesticides,” “testicular health,” “male fertility,” “oxidative stress,” endocrine disruption,” “natural products,” and “antioxidants” were used in an online search conducted between June and November of 2024 to identify research publications and reviews published in English over the previous decade. They were located in various medical databases, including ScienceDirect and PubMed. The reviews and original articles were accorded the most weight.

Generally speaking, it is yet unknown what possible underlying processes underline the reprotoxic effects of pesticides. Even though a lot of research has shown how harmful pesticides are to different organs, a thorough analysis that focuses on the molecular pathways by which they affect male fertility is still needed. The detrimental effects of pesticides on humans and animals, which can have an impact on male fertility, are highlighted in this review along with the processes underlying these effects. We also point out knowledge gaps that need more research.

## Chemistry of insecticides

Pesticides are chemical groups that are used to eradicate pests encompassing fungicides, insecticides, and herbicides (Doble and Kumar 2005; El Nemr et al. 2012). Insecticides are classified as pyrethroids, organochlorines, organophosphates and carbamates, insect growth regulators (IGRs), and neonicotinoids.

### Pyrethroids

**Fig. 1:**
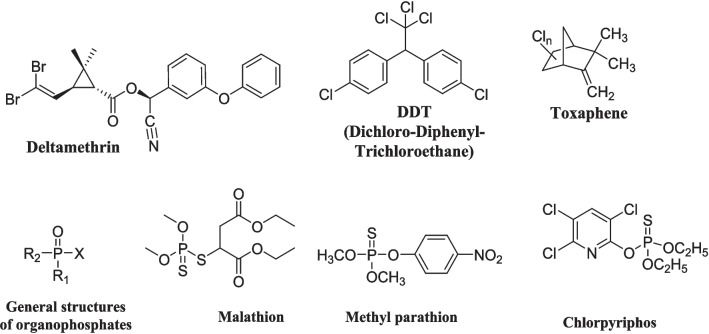
Chemical structure of representative examples of pyrethroids, organochlorines, and organophosphates

Pyrethroids are synthetic chemicals similar to the natural pyrethrins (produced by the flowers of pyrethrums; *Chrysanthemum cineraria folium* and *C. coccineum*), which target voltage-gated sodium channels in the insect nervous system (Soderlund et al. 2002; Taniguchi et al. 2019; Andersen et al. 2022). Many pyrethroids are 2,2-dimethyl cyclopropane carboxylic acid derivatives, likewise chrysanthemic acid, that is esterified with alcohol (Galadima et al. 2021). However, the cyclopropyl ring does not occur in all pyrethroids, such as Fenvalerate (Organization 1990). Some pyrethroids, etofenprox, for instance, lack the ester bond and an ether bond is used instead. Selaflofen is another pyrethroid that contains a silicon atom instead of an ester (Taniguchi et al. 2019). Pyrethroid chemistry is classified as type 1 or type 2, depending on the alcohol substituent in the molecule. The type 1 group lacks the alpha-cyano group and is broadly defined as pyrethroids containing decyano nonphenoxybenzyl, decyano-3-phenoxybenzyl, or other alcohols (Eljarrat 2020). Type 2 pyrethroids precisely contain an alpha-cyano-3-phenoxybenzyl alcohol (-CN), which enhances the insecticidal activity almost tenfold and makes them notably more toxic (Osman 1989; Ahamad and Kumar 2023). Natural pyrethrins are six ester compounds that result from the condensation of one alcohol and acid, which may be pyrethric acid or chrysanthemic acid (Crombie 1980). The main pyrethroid products are bifenthrin, deltamethrin, permethrin, cypermethrin, cyfluthrin, *lambda-cyhalothrin,* and others, whereas bifenthrin refers to a synthetic pyrethroid insecticide that is widely used for pest control in various agricultural and residential settings (Fig. 1) (Ahamad and Kumar 2023).

### Organochlorines

The nervous system is the principal site of action of organochlorines, where they bind to the sodium channel and cause delayed Na-inactivation, which in turn leads to a prolonged delay in Na-inactivation and subsequent interference with nerve impulse functions (Lalah et al. 2022).

The organochlorine insecticides are classified into three major classes, including DDT *and its* analogs, the benzene hexachloride (BHC) isomers*,* and the cyclodiene compounds, respectively. DDT (dichloro-diphenyl-trichloroethane, p,p′-DDT), its isomer impurities (o,p′-DDT and o,o′-DDT), and the formed metabolites in the organisms or the environment (DDE) are more toxic, potent, and persistent (Ware and Whitacre 2004; Simon 2011). γ-Hexachlorocyclohexane (γ-HCH) (Lindane®) exhibits significant insecticidal activity and is broadly used in agriculture. Cyclodiene compounds are cyclic hydrocarbon insecticides that include aldrin, dieldrin, chlordane (two isomers, α and β-chlordane), heptachlor, endrin, endosulfan, chlordecone, mirex, and toxaphene. (Ware and Whitacre 2004; Simon 2011) Toxaphene is a mixture of hundreds of various chemicals with a major toxic component called heptachlorobornane (Fig. 1) (Saleh 1983).

### Organophosphates

Organophosphates (Ops) are among the most widely utilized pesticides worldwide. This chemical group resulted from an esterification reaction between phosphoric acid or thiophosphoric acid and alcohols (Hassaan and El Nemr 2020). In addition to their pesticidal activity, Ops represents the essential components of the neurotoxic nerve agents (like sarin, soman, and tabun) and also has a significant application in the production of plasticizers, solvents, and flame retardants (Aroniadou-Anderjaska et al. 2023). Their pesticidal potentiality is due to their capability to inhibit irreversibly the neural acetylcholinesterase enzyme (AChE), the enzyme that is involved in the breakdown of acetylcholine (Ach) neurotransmitter in the synapse (Ranjan et al. 2022).

Ops form covalent bonds with the serine hydroxyl group within the catalytic active site of AChE, producing a phosphorylated enzyme (Ranjan et al. 2022) (Vale and Lotti 2015). The produced phosphorylated enzyme complex is highly stable, leading to the irreversible inactivation of AChE for several hundred hours (English and Webster 2012). This results in the elevation of Ach levels, overstimulation of nicotinic receptors in the central nervous system, and eventually insect death (Aroniadou-Anderjaska et al. 2023). Noteworthy, Ach is conserved among species, and thus the enormous use of Ops pesticides poses a potential toxicity to non-target species like humans. In mammals, acute or chronic exposure to ops is followed by overstimulation of both nicotinic and muscarinic receptors and a series of clinical symptoms accompanied by cholinergic toxicity (Aroniadou-Anderjaska et al. 2023).

The general structure of Ops (Fig. 1) has a central pentavalent phosphorus atom bound to an oxygen or sulfur atom (through a covalent double bond), two alkyl groups either methoxy or ethoxy groups (R_1_ and R_**2**_) and X as a leaving group (Ranjan et al. 2022). The leaving group (X) has a vital function in their toxicity and potentiality (it is replaced with the serine oxygen atom through a nucleophilic substitution reaction producing an inactive phosphorylated enzyme) (Ranjan et al. 2022). Some Ops have a thiono group (P = S linkage) and require metabolic activation by cytochrome P450 to the active oxon form (P = O) to inhibit the targeted AChE. They are generally classified into three groups (Jepson 2013): (1) aliphatic Ops (Fig. 1) that comprise the oldest compounds like malathion, acephate, and dichlorvos; (2) phenyl Ops (Fig. 1) that exhibit large toxicity to mammals including methyl parathion and fenitrothion; and (3) heterocyclic Ops (Fig. 1) like chlorpyriphos and pyrazophos.

### Carbamates

**Fig. 2:**
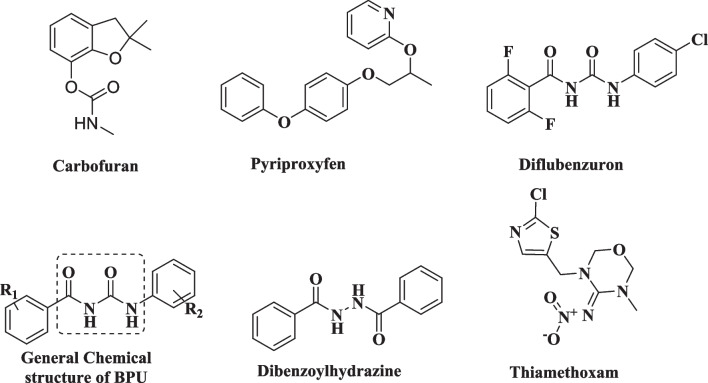
Chemical structure of representative examples of carbamates, insect growth regulators and neonicotinoids

Carbamates are widely used as pesticides and also have significant therapeutic applications, including the treatment of Alzheimer’s disease, glaucoma, and myasthenia gravis (King and Aaron 2015). Carbamates are esters of N-methyl carbamic acid and were primarily synthesized as newly developed insect repellants, but their pesticidal activity was discovered rapidly (Kumar et al. 2022). Fenobucarb, carbaryl, and carbofuran (Fig. 2) are examples of pesticides containing the carbamate functional group. Carbamates exert their pesticidal activity by reversibly inhibiting the acetylcholinesterase enzyme. Upon binding to AChE, they undergo two hydrolytic steps, forming covalent bonds with the hydroxyl group of a serine residue (within the catalytic active site of AChE) and producing a carbamoylated AChE at the neuronal synapses. The produced enzyme complex is stable to hydrolysis, leading to reversible inactivation of AChE for more than 4 h (English and Webster 2012).

### Insect growth regulators

Insect growth regulators (Insect growth disruptors) include three classes; Juvenile Hormone analogs (JHA), also named JH mimics or juvenoids, Chitin synthetase inhibitors (CSIs) that are mainly benzoylphenyl urea (BPU), and Ecdysone agonists (EAs) (Pener and Dhadialla 2012).

JHA are insecticides with a major role in controlling adult pests and disease vectors by inducing larval period extension in most insects, killing pupae but not larvae in some insects, and blocking the death of larval cells, but the effect on the proliferation and differentiation of imaginal cells is variable. Methoprene and pyriproxyfen are currently used in large quantities to control mosquitoes (Parthasarathy and Palli 2021). CSIs are widely used in integrated pest management (IPM) and insecticides. Chitin synthesis inhibitors include mainly BPU, which are widely used in integrated pest management (IPM) and insecticide (Sun et al. 2015). Diflubenzuron is the first commercial product (Degheele et al. 1993), while Novaluron is a second-generation BPU (Fig. 2) (Hwang et al. 2022).

The BPU can be divided into three parts for analysis of the SARs, namely, the benzoyl ring, the aniline ring, and the urea bridge, as each moiety affects the overall expression of larvicidal activities.

(i) Benzoyl ring substitutions such as a 2-Cl, 2,6-diCl, or 2,6-diF substitution are essential for optimal activity, with the 2,6-diF substitution pattern being most effective. The bioisosteric replacement of the benzoyl ring (A) with aromatic heterocycles or cyclic alkyl substituents maintains larvicidal activity yet lowers that of benzoyl moieties.
(ii) Urea bridge moiety replacement with thiourea worsens larvicidal activity worse than that of the urea derivatives. N-Alkylation was found to improve the poor solubility of BPUs in organic solvents but with lower larvicidal activity in comparison with their parent structure.
(iii) The aniline moiety optimization depends on different functional groups, and pests have different SARs. Generally, bulky groups at the 2,6-position of the aniline moiety reduce larvicidal activities, while electron-withdrawing groups at the 4-position of the aniline moiety enhance the insecticidal effect. Replacing the aniline ring with other aromatic heterocycles may cause a slight reduction in activity (Fig. 2) (Sun et al. 2015).

In insects, the ecdysone receptor regulates the metamorphosis process. The commercialized insecticide tebufenozide kills some lepidopteran and coleopteran pests by interfering with the normal molting process (Feng et al. 2023). These compounds are synthetic analogs of natural ecdysone. When applied to insects, it kills them by the formation of defective cuticles. The development processes are accelerated, bypassing several normal events, resulting in an integument lacking scales or a wax layer (Fig. 2) (Guo et al. 2023).

### Neonicotinoids

Neonicotinoids act selectively on insect nicotinic acetylcholine receptors (nAChRs), thus mediating the fast excitatory synaptic transmission in the insect’s central nervous system (Jeschke et al. 2011)**.** The neonicotinoids currently on the market include the cyclic compound, with a five-membered ring system, such as imidacloprid, and with a six-membered neonicotinoid thiamethoxam (Syngenta) and a noncyclic compound, acetamiprid (Fig. 2) (Jeschke et al. 2011).

## Pharmacokinetics of insecticides

The pharmacokinetics of insecticides pertains to the examination of their absorption, distribution, metabolism, and excretion within living organisms. Comprehending these processes is essential for assessing their toxicological effects and risks to human health and the environment.

Insecticides must first be taken into the organism to manifest their poisonous effects (Timchalk 2006). The primary pathways of absorption for pesticides are ingestion, cutaneous contact, and inhalation. Upon accessing the body, the pesticide is disseminated to multiple tissues (Dix 2010). Organophosphates, including chlorpyrifos and diazinon, are readily absorbed and exhibit their harmful effects by competing for metabolic routes, hence modifying their pharmacokinetic profiles when administered concurrently. This indicates that pharmacokinetic interactions among pesticides can affect their toxicity, particularly in mixed exposures prevalent in environmental and occupational conditions. These insecticides inhibit acetylcholinesterase (AChE), an enzyme essential for nerve activity, after being converted into active metabolites (e.g., oxons) that permanently bind to acetylcholinesterase (AChE), resulting in the buildup of acetylcholine at synapses and consequent paralysis of the insect (Poet et al. 2004; Timchalk et al. 2005).

Pesticides experience metabolic conversions, predominantly in the liver, facilitated by cytochrome P450 (CYP) enzymes. Certain pesticides function as both substrates and inhibitors of these enzymes, affecting their degradation and that of other substances. These metabolic pathways provide a dual function: they convert certain pesticides into more harmful metabolites and detoxify others (Hodgson et al. 1995). Chlorpyrifos is converted into chlorpyrifos-oxon, the active molecule that inhibits AChE (Timchalk and Poet 2008). Moreover, neonicotinoids, a contemporary category of insecticides, are metabolized in manners that yield comparatively little toxicity to mammals, attributable to their advantageous selectivity for insects rather than vertebrate nicotinic acetylcholine receptors (Tomizawa and Casida 2005).

The last phase of the pharmacokinetic cycle is elimination, during which the insecticide or its metabolites are expelled from the human body. Almost all pesticides are excreted through urine or stools. The elimination rate from the body may fluctuate based on the chemical characteristics of the pesticide. Organophosphorus insecticides are excreted through urine, and pharmacokinetic models assist in estimating their dosage depending on urinary excretion data. Research employing physiologically based pharmacokinetic models has demonstrated that pesticide excretion rates can forecast long-term exposure danger (Lu et al. 2010). The chemical’s half-life determines the elimination rate, impacting both its effectiveness and its tendency to accumulate in the body. For instance, pyrethroids, a category of synthetic insecticides, demonstrate differing clearance rates based on their chemical composition. Pyrethroids with delayed clearance rates are generally more harmful to insects, as they sustain elevated concentrations at their sites of action for extended durations (Soderlund 1983).

Chlorpyrifos metabolites, including 3,5,6-trichloro-2-pyridinol, are identified in urine and blood after exposure in both human and animal investigations, with pharmacokinetic models facilitating the estimation of the chemical’s pharmacokinetic parameters. Research indicates that although humans and rats metabolize chlorpyrifos comparably, humans often exhibit reduced efficiency in generating the active metabolite, chlorpyrifos-oxon, compared to rats, perhaps elucidating species-specific differences in toxicity (Timchalk et al. 2002).

## Pharmacological properties of insecticides

Although pesticides are categorized based on their chemical structure, their pharmacological effects are largely similar. The four main types include organophosphorus compounds, neonicotinoids, pyrethroids, and insect growth regulator inhibitors. A common adverse effect of these chemicals is endocrine disruption, as they are considered potential endocrine disruptors, mainly affecting the reproductive hormonal pathways (Sweeney et al. 2000).

Organophosphorus compounds (OP) inhibit acetylcholinesterase, leading to increased levels of acetylcholine, which causes acute cholinergic syndrome. The enzyme inhibition results in acetylcholine accumulation, leading to poisoning symptoms in both animals and humans (Joshi and Sharma 2011). Exposure to these compounds negatively impacts fertility, growth, and development in both males and females (Woodruff et al. 2010). In males, OP pesticide exposure has been linked to poor semen quality, reduced sperm count, seminal volume, and motility (Woodruff et al. 2010). Numerous studies show that OP-induced reactive oxygen species can damage reproductive tissues, playing a role in testicular pathophysiology, likely through the inhibition of enzymes involved in DNA synthesis and membrane polyunsaturated fatty acid metabolism (Nagda and Kumar Bhatt 2011). This also reduces the activity of glutathione (GSH), GSH peroxidase (Mandal and Das 2004), and antioxidant vitamins (Yousef 2010).

Neonicotinoids, related to nicotine, act on nicotinic acetylcholine receptors (nAChRs) in the central and peripheral nervous systems, overstimulating these receptors and causing paralysis and death at high levels (Costas-Ferreira and Faro 2021). While mammalian nAChRs are similarly affected, these compounds have a lower affinity for them but have still been shown to impair reproduction in mammals (Bal et al. 2012b).

Nicotine exposure, whether acute or chronic, induces oxidative stress, harming reproductive organs in both animals and humans (Jain and Flora 2012). Synthetic pyrethroids, widely used pesticides, exhibit relatively low toxicity in humans compared to other pesticide classes like organochlorines and carbamates (Thatheyus and Selvam 2013). However, their negative impacts on humans and animals include nephrotoxicity (Xu et al. 2015), hepatotoxicity (Xu et al. 2015), cardiotoxicity (Vadhana et al. 2013), immunotoxicity (Skolarczyk et al. 2017), and neurotoxicity (Nasuti et al. 2013). Pyrethroids induce oxidative stress, increasing the activity of SODs, glutathione S-transferases, and CAT while reducing GPx activity, GSH levels, and certain interleukins in plasma (Xu et al. 2015). They target voltage-gated sodium channels in nerve and muscle cells, preventing the channels from closing, which overstimulates the nervous system and kills the insect (Field et al. 2017). Insect growth regulator inhibitors (IGRs) disrupt biochemical pathways absent in humans, making them relatively safe but not without risks (Bell 2014). IGRs can cause organ toxicity, endocrine disruptions, and teratogenic effects in animals and humans, potentially leading to reduced body weight and testicular damage, thus interfering with spermatogenesis (Bell 2014).

## Potential mechanisms of testicular injury due to insecticides

### Oxidative stress

It is now widely acknowledged that oxidative stress plays a significant role in testicular injury, especially when exposed to environmental pollutants like pesticides (Asadi et al. 2017). Testicular tissues have been found to exhibit elevated amounts of reactive oxygen species (ROS) after exposure to insecticides like imidacloprid and endosulfan. This elevation can be caused by mitochondrial malfunction, which results in an overabundance of free radicals, or by direct cellular injury (Asadi et al. 2017; Keles et al. 2022). Enzymes such as catalase and superoxide dismutase (SOD) are part of the testis’ abundant antioxidant system. Such systems can be compromised by pesticide exposure, which lessens their ability to counteract ROS and causes oxidative damage (Turner and Lysiak 2008).

Exposure to pesticides has been linked to Significant degradation of germ cells and seminiferous tubules revealed by histological investigations of testicular tissues. According to a previous study, the testes of rats given Imidacloprid showed signs of extreme oxidative stress, including elevated lipid peroxidation and cell death (Hussein et al. 2018; Keles et al. 2022). Increased production of ROS by insecticide showed a dramatical impact on male infertility through impaired spermatogenesis and reduced sperm motility along with DNA damage and changes in sperm shape (Turner and Lysiak 2008; Asadi et al. 2017).

**Fig. 3:**
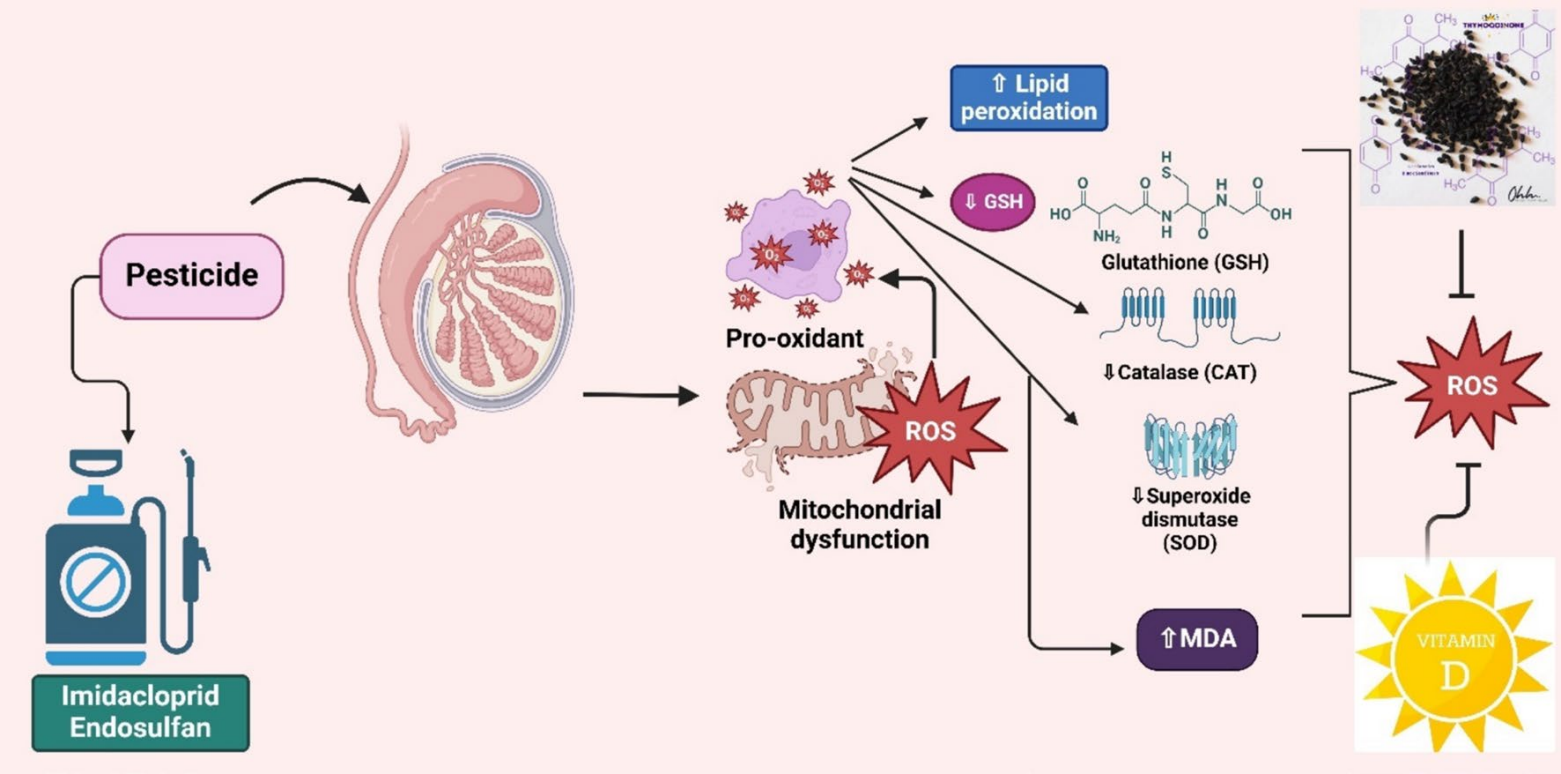
Pesticide-induced oxidative stress disrupts testicular antioxidant systems. Pesticide elevates ROS levels, and damages cellular structures, with protective roles identified for compounds such as vitamin D and thymoquinone

Studies revealed that some compounds, including vitamin D and thymoquinone, have demonstrated potential in protecting testicular tissues from insecticide-induced oxidative damage. To prevent testicular damage, these substances may lower levels of ROS or increase the activity of antioxidant enzymes (Hussein et al. 2018) (Fig. 3).

### Endocrine disruption

#### Endocrine disruptors via AR antagonism

Pesticides such as synthetic pyrethroids, organophosphates, carbamates, and organochlorines are known as endocrine-disrupting chemicals (EDCs) (Ghosh et al. 2022). They are inducting damage in the male reproductive system through hormone imbalance in the endocrine system. (Diamanti-Kandarakis et al. 2009; Kahn et al. 2020). Due to their similar structure to reproductive steroid hormones, they could induce direct harmful effects on the endocrine system by binding to its receptors and antagonizing its effect (Oliva et al. 2001). Furthermore, they can affect the release of testosterone and gonadotropin action (Warner et al. 2020).

**Fig. 4:**
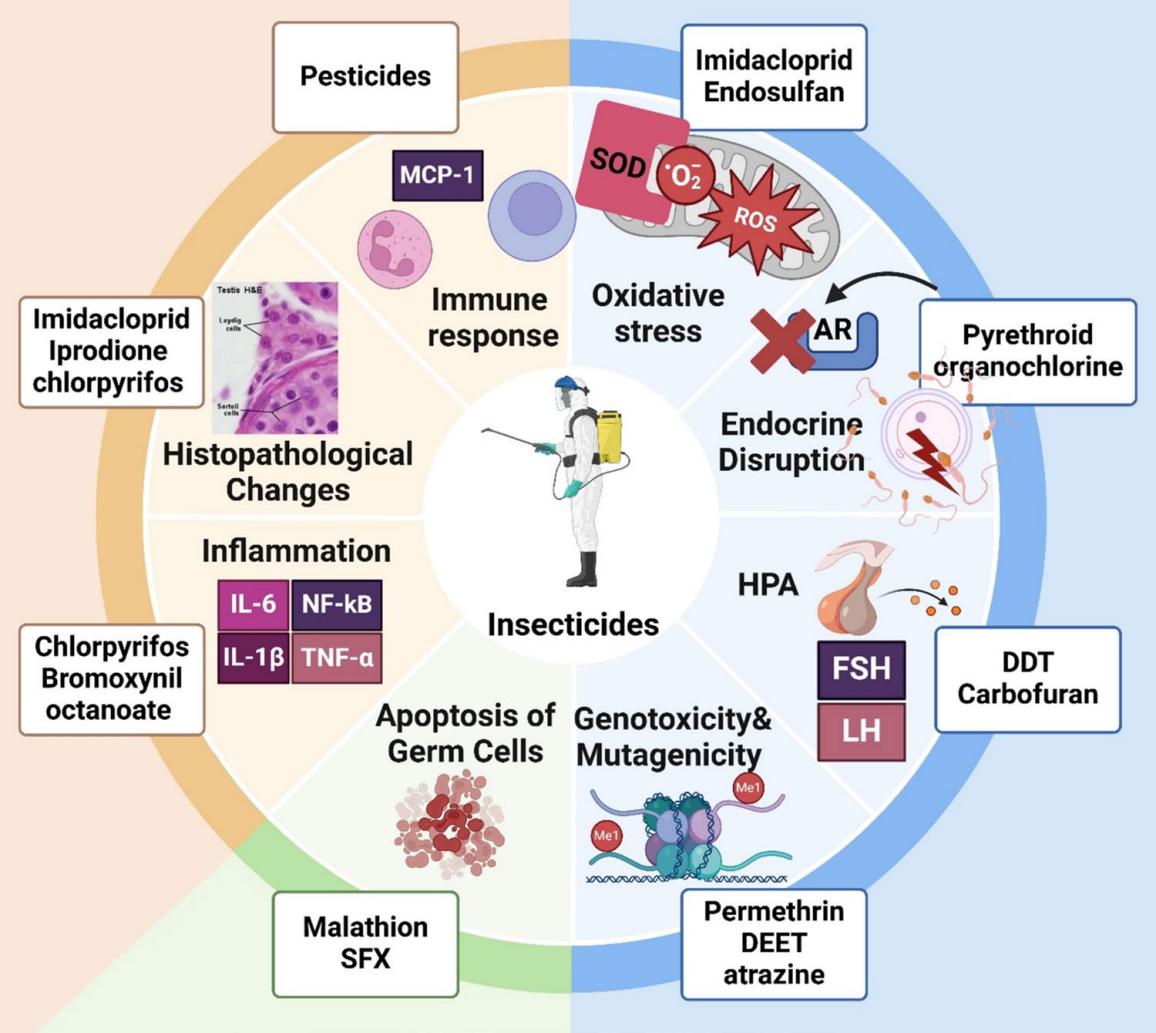
Pesticides cause testicular toxicity via oxidative stress, endocrine disruption, and cellular damage, impairing spermatogenesis and sperm quality

Dichloro-diphenyl-trichloroethane (DDT), dichloro-diphenyl-dichloroethylene (DDE), dichloro-diphenyl-dichloroethane (DDD), and methoxychlor are organochloride insecticides proved in vivo inhibition of androgen receptor (AR)-dependent transcriptional activity (Jayaraj et al. 2016). AR antagonism has an infertility consequence and negatively influences spermatogenesis (Warner et al. 2020). Because of the similarity of cypermethrin and deltamethrin to the AR native ligand; testosterone, they form stable AR–ligand complexes with good-quality bonds via several molecular interactions. A hydrogen bond, pi–pi interactions, and salt bridge contribute to the stability of these complexes (Jeong et al. 2023; Sheikh et al. 2023). Many animal studies discussed the negative effect of organophosphate on the reproductive system also through androgen receptor antagonism (Tamura et al. 2001; Hu et al. 2023). Chlorpropham, a carbamate ester herbicide, also has endocrine-disrupting effects by suppressing the dimerization of ligand-bound ARs in the cytoplasm (Jeong et al. 2023) (Fig. 4).

#### Endocrine disruptors via the hypothalamic–pituitary testicular pathway

Pesticides affect the male reproductive system by interrupting the synthesis and release of gonadotropin, luteinizing hormone (LH), and follicle-stimulating hormone (FSH). Normally, gonadotropin-releasing hormone (GnRH) is released by the hypothalamus gland. This stimulates the synthesis and secretion of hormones (Ghosh et al. 2022). DDT has adversely affected the male reproductive system due to the disruption of the hypothalamic-pituitary–testicular axis in addition to the previously discussed direct interaction with the sex steroid receptors (Ghosh et al. 2022). A decreased level of testosterone production by the testes is induced by the impairment of negative feedback exerted by the steroid on the hypothalamic-pituitary axis. Thus, it increases a gonadotrophin level which leads to increased serum levels of LH and FSH (Rhouma et al. 2001; Verma and Mohanty 2009), when assessing the pituitary functions in human (Şimşek et al. 2019) and animal (Uzun et al. 2009). Study after acute and chronic exposure to organophosphorus (OP), decreased plasma FSH, LH, and level GH were observed.

Carbofuran, which is classified as a carbamate pesticide, induces endocrine disruption via reversible inhibition of the neurotransmitter acetylcholine (AChE) through carbamylation. Accumulation of AChE in brain synapses and neuromuscular junctions eventually leads to decreased serum testosterone concentration and sperm count (Moreira et al. 2021, 2022). Also, carbamate pesticides interrupt the hypothalamic–pituitary–thyroid axis (HPT axis) pathway, which leads to deficient GnRH, LH, and/or FSH levels, thus compromising steroidogenesis and spermatogenesis (Pant et al. 1995; Sobhy et al. 2017). Another suggested mechanism regarding carbamate’s effect on male fertility is carbamate negatively influences the kisspeptin signaling. kisspeptin peptides bind to its receptors in the testis to regulate the fertilizing capacity of spermatozoa. This leads to a significant decrease in serum testosterone levels (Moreira et al. 2022) (Fig. 4).

### Genotoxicity and mutagenicity

Pesticides can induce changes in DNA structure, leading to gene mutations that could be inherited from father to child (Giambò et al. 2021).

#### DNA methylation

DNA methylation with methyl groups, through DNA methyltransferases (DNMTs), induces a modification that blocks access of transcription factors to genes. Methylated DNA could be inherited by offspring and later generations, leading to transgenerational effects (Warner et al. 2020; Giambò et al. 2021). A conducted study on agricultural workers, who exposed to permethrin and DEET, observed DNA methylation alterations are found in the sperm (Howard et al. 2016; Thorson et al. 2020). Gestational exposure (F1) to atrazine herbicide led to increased testis disease in the F3 generation (McBirney et al. 2017). Each exposed generation has a different methylated region (F1 as embryo; F2 as germ cells; F3 no direct exposure).

#### Histone modification

Histones are vital proteins for chromatin formation in the nucleus of a cell (Fyodorov et al. 2018). Originally, DNA winds around histone; then, it unwinds for transcription. Histones can be modified through methylation and acetylation which limits the accessibility of genes (Warner et al. 2020).

It is suggested that carbendazim (CBZ) (Liu et al. 2019) and chlorothalonil (Zhang et al. 2019), a widely used broad-spectrum carbamate fungicide, impair mouse spermatogenesis through histone modification and DNA methylation (Liu et al. 2019) in addition to its endocrine-disrupting effects.

#### Non-coding RNAs

Non-coding RNAs, including small microRNAs (miRNAs), are important regulators of post-transcriptional gene expression. Sperm mRNAs could be potential markers of male fertility (Zhang et al. 2019). Altered miRNAs are associated with epigenetic regulation of carcinogenesis (Warner et al. 2020). Recent studies illustrated the possible alterations in sperm through DNA methylation, non-coding RNA, and histone retention when exposed to DDT (Skinner et al. 2018) and its metabolites (Frigo et al. 2002) and DDE exposure (Lu et al. 2024).

### Histopathological changes

The testis in its mature state is comprised of densely convoluted tubules containing germ cells and Sertoli cells. These tubules are divided by an interstitium that contains macrophages, capillaries, Leydig cells, an ultrafiltrate rich in testosterone and protein, and a supportive stroma. The seminiferous epithelium is composed of Sertoli cells positioned at the base, which provides support to many populations of germ cells that are maturing simultaneously (Whitney 2012). There is substantial evidence that Insecticide exposure causes histopathological alterations in testicular tissue, which in turn compromises reproductive health. Sertoli cell-only pattern, calcification, disorganization, hypo-spermatogenesis, hypoplasia of germ cells, spermatic cell arrest, sloughing, and fibro-hyalinized tubules are histopathological alterations that can be seen in testes due to various toxicants (Shojaeepour et al. 2021).

Other histopathological changes may include thickening and hyalinization of the Tunica Propria surrounding the seminiferous tubules, along with significant lymphocyte infiltration, which is consistent with inflammatory responses. Damage to Seminiferous Tubules, shedding of the spermatogenic epithelium, alterations in the blood-testis barrier (BTB), and changes in Leydig cells, collectively, can result in impaired spermatogenesis and testicular dysfunction (Johnson 2014; Vidal and Whitney 2014; Ly et al. 2023).

It was found that neonatal rat exposure to imidacloprid (IMD) resulted in male infertility by damaging the testicles, lowering testosterone production, and raising oxidative stress levels (Sardar et al. 2023), such lowering of testosterone levels occurs via degeneration of Leydig cells and reduction of their number (Lonare et al. 2016) or indirectly as an acetylcholine receptor agonist, it has a nicotine-like inhibitory activity on LH release from the pituitary gland (Mohamed et al. 2017; Abd-Elhakim et al. 2021). Also, a decrease in tubular diameter and epithelial height in groups exposed to IMD for 26 days, while there was an increase in lumen diameter and interstitial space, which indicates a compromise in the process of spermatogenesis (Sardar et al. 2023). Moreover, IMD histopathological observations included cytoplasmic granulation and hypertrophy (Lonare et al. 2016).

The relative weights of the gonads and auxiliary glands (SVs and prostate glands) were significantly reduced. This could be because the exposure to IMD caused the seminiferous tubules to atrophy and the quantity of Leydig cells to decrease (Mohamed et al. 2017). One sensitive indicator of chemical-induced reproductive toxicity is the weight of reproductive organs (Abd-Elhakim et al. 2021) (Zidan 2009).

Iprodione and chlorpyrifos IPR and CPF-exposed adult rats had testicular tissue changes with spermatogenesis disruption. Diminished seminiferous tubules had desquamated germ cells, few sperm cells, and minimal spermatid debris. Also, epididymal tubule blood vessel congestion and mild to severe epithelial degradation and cytoplasmic vacuolation. The prostate gland showed epithelial hyperplasia in most acini with papillary projections, and the seminal vesicles showed glandular atrophy with destroyed folds and limited fluid (Abd-Elhakim et al. 2021). Additionally, a reduced testicular weight was observed and attributed to a decline in tubular size (Hassan et al. 2021). Regarding malathion, alteration of the epididymitis tissue morphology and compromise the sperm maturation process were observed (Abd-Elhakim et al. 2021).

SFX on the other hand caused testicular degeneration, and diminished layers in some seminiferous tubules which showed dilatation with eosinophilic fluid in a rat model. Upon increasing the dose of SFX, expanded testicular damage occurred, including degeneration, dilatation, and hypereosinophilic disordered vacuolated cells in most seminiferous tubules. Additionally, interstitial edema with moderate Leydig cell hyperplasia was seen. Also, a decrease in the concentration of sperm in the epididymal lumen was found along with a buildup of large spermatid cells. Collectively, pesticides have detrimental effects on the reproductive system because of their electrophilic attack on cells (Mohamed et al. 2022).

### Apoptosis of germ cells

Various germ cells in testicular tissue include spermatogonia, spermatocytes, and spermatids, all of which differ in ploidy, morphology, and sensitivity to adverse impacts, including insecticides. Spermatogonia are the only proliferative cells in the seminiferous epithelium outside the blood-testis barrier. Due to their mitotic activity and direct exposure to the seminiferous tubule interstitial ultrafiltrate, these diploid cells are particularly susceptible to cytotoxic chemicals (Whitney 2012). Apoptosis is crucial for preserving testicular homeostasis during spermatogenesis. A significant fraction of developing spermatogenic cells is thought to undergo apoptosis. When normal testes are evaluated histologically, only a small fraction of cells appear to be apoptotic (Yu et al. 2014). In all testicular specimens, a strong association was seen between the quantity of apoptotic germ cells and the total amount of sperm (Ilyas et al. 2016). A study of the effect of deltamethrin on the apoptosis of germ cells attributed such apoptotic events to the disturbance in oxidative stress and decreased levels of eNOS (Yu et al. 2014). Also, IMI compromises testicular tissue by reducing the regeneration of type A spermatogonia, which is crucial for spermatogenesis, it induces oxidative testicular damage followed by DNA fragmentation and apoptosis of spermatogonia (Mohamed et al. 2017).

IPR and CPF combined exposure was linked to a considerable decline in testicular biomarkers such as ACP and SDH, which is indicative of a decline in testicular energy metabolism, ability to preserve normal physiological and metabolic processes, as well as spermatogenesis, all of which could be attributable to an increased rate of apoptosis (Abd-Elhakim et al. 2021; Hassan et al. 2021).

Moreover, malathion exposure causes an inflammatory cascade that increases neutrophil and macrophage populations in the epididymis (Erthal et al. 2020). Exposure to malathion for longer periods resulted in a reduction in spermatozoal count, count of testicular spermatids, and motility of sperms, besides an increase of apoptotic markers highlighting a prominent role in the promotion of apoptosis in testicular tissue (Selmi et al. 2015). Malathion has been shown to disrupt seminiferous epithelial proliferation by inhibiting steroidogenesis, resulting in germ cell death (Penna-Videau et al. 2012), Malathion toxicity in testicular cells was investigated for apoptosis, and the results revealed the presence of a variety of apoptotic characteristics in malathion-treated groups, including pyknosis, vacuolization, empty spaces, tubular degeneration, and condensed or fragmented chromatin in testicular germ cells (Bhardwaj et al. 2018) (Fig. 4).

SFX-exposed rats showed a high percentage of sperms with damaged DNA when compared to an unexposed group; also, higher levels of serum MDA were observed which correlated with infertility attributes and an increased sperm apoptosis frequency (Mehraban et al. 2019; Mohamed et al. 2022). Thus, it could be proposed that insecticides inhibit spermatogenesis, as a result of the germ cell apoptosis (Park et al. 2020).

### Inflammation and immune response

Exposure to various insecticides can significantly impact testicular health and provoke inflammatory responses, leading to detrimental effects on male reproductive function. Some of the key inflammatory markers impacted by such exposure are as follows.

#### Tumor necrosis factor-alpha (TNF-α)

Exposure to specific insecticides, such as chlorpyrifos and heptachlor epoxide, results in elevated tumor necrosis factor-alpha (TNF-α) levels and increased production, suggesting a direct impact on inflammatory pathways (Gangemi et al. 2016). The elevation of TNF-α following insecticide exposure may occur through the activation of signaling pathways such as nuclear factor-kappa B (NF-κB). This pathway is known to regulate the expression of numerous inflammatory cytokines, including TNF-α, thereby contributing to the inflammatory response observed in tissues affected by pesticide exposure (Lopes-Ferreira et al. 2023).

The increase in TNF-α levels due to insecticide exposure may impair immune function and contribute to immunotoxicity leading to chronic inflammation, which has been associated with various health issues, including cancer development and reduced immune surveillance capabilities (Gangemi et al. 2016). This cytokine is known to promote the recruitment and activation of neutrophils, which release additional inflammatory mediators and ROS. This cascade can exacerbate tissue damage and inflammation within the testes, potentially impairing spermatogenesis and overall reproductive health (Lopes-Ferreira et al. 2023; Petricca et al. 2023). Also, the persistent elevation of TNF-α in response to pesticide exposure can contribute to chronic inflammation. Chronic exposure to certain pesticides leads to significant histopathological changes in testicular architecture, including degeneration of seminiferous tubules and disruption of normal spermatogenic processes (Hassan et al. 2021)***.*** Furthermore, elevated TNF-α levels play a role in neoplastic transformation at sites of chronic inflammation. For example, TNF-α can induce DNA damage when combined with other factors like estrogens, raising concerns about the potential carcinogenic effects of prolonged pesticide exposure (Gangemi et al. 2016) (Fig. 4)*.* In conditions such as experimental autoimmune orchitis, autoimmune aggression to the testes characterized by the presence of specific antisperm autoantibodies, TNF-α released by testicular macrophages leads to a significant rise in apoptotic germ cells, particularly those expressing TNF receptor 1 (TNFR1). Approximately 60% of TNFR1-positive germ cells were found to be apoptotic in these models, indicating a direct detrimental effect on spermatogenesis (Suescun et al. 2003; Theas et al. 2008). TNF-α disrupts normal testicular functions, including testosterone production. It inhibits testosterone secretion from Leydig cells, both at basal levels and when stimulated by human chorionic gonadotropin. This inhibition is linked to decreased expression of key enzymes involved in testosterone biosynthesis (Suescun et al. 2003; Lysiak 2004). It also affects the structural integrity of the blood-testis barrier (BTB), reversibly disrupting the junctions between Sertoli and germ cells, impairing their adhesion within the seminiferous epithelium. This disruption can hinder the migration of spermatocytes during critical stages of spermatogenesis (Li et al. 2006).

#### NF-кB

The inflammatory response is another critical factor in insecticide-induced testicular injury. Exposure to certain pesticides can trigger inflammatory pathways that exacerbate tissue damage. For example, bromoxynil octanoate has been shown to induce inflammation and apoptosis in testicular tissues through modulation of the nuclear factor kappa-light-chain-enhancer of activated B cells (NF-кB) pathway. This inflammatory response can lead to further oxidative damage and impair spermatogenesis (El-Nagar and Elsisi 2023)***.*** The bromoxynil octanoate herbicide exposure triggered an inflammatory response characterized by increased expression of TNF-α and IL-6. This inflammation is linked to the activation of the NF-кB signaling pathway, which plays a crucial role in mediating inflammatory responses (El-Nagar and Elsisi 2023).

The NF-κB pathway also regulates apoptosis in testicular tissue, particularly under stress conditions. NF-κB is a key regulator of various pro-inflammatory cytokines, such as TNF-α and IL-6, which can further activate apoptotic pathways. The expression of these cytokines can lead to increased apoptosis in germ cells (Pentikäinen et al. 2002; Teng et al. 2012)***.*** NF-κB activation in Sertoli cells has been shown to exert proapoptotic effects on germ cells. During testicular stress, the levels of nuclear NF-κB increase, which correlates with heightened apoptosis of male germ cells (Pentikäinen et al. 2002)***.*** NF-κB is known to regulate specific genes critical for spermatogenesis. For instance, it positively regulates the expression of LRWD1, a testis-enriched gene essential for male germ cell function. The presence of an NF-κB binding site in the LRWD1 promoter allows NF-κB to enhance its transcription, indicating that NF-κB directly influences genes that are crucial for sperm development and maturation (Teng et al. 2012). In the testis, NF-κB is activated in a stage-specific manner during spermatogenesis. It is present in Sertoli cells and germ cells at different stages, suggesting that NF-κB plays a role in modulating gene expression according to the developmental stage of the sperm cells. This stage-specific activation is critical for coordinating the complex processes involved in sperm production (Pentikäinen et al. 2002).

Exposure to imidacloprid has been shown to induce significant testicular damage characterized by oxidative stress, inflammation, and apoptosis in male rats. This insecticide disrupts normal testicular function by increasing levels of malondialdehyde (MDA) and decreasing antioxidant enzyme activities such as superoxide dismutase (SOD) and catalase. Histopathological examinations revealed degenerative changes in seminiferous tubules, leading to impaired spermatogenesis (Hussein et al. 2018) (Fig. 4). Elevated NF-κB activity can exacerbate inflammatory responses, contributing to conditions like testicular heat stress, which has been shown to increase germ cell apoptosis and disrupt normal testicular function (Mathur et al. 2011; Hu et al. 2021). Inflammatory pathways mediated by NF-κB are linked to poor semen quality. Genetic polymorphisms in the NF-κB1 gene have been associated with variations in semen parameters among men, suggesting that NF-κB activity may influence male fertility (El‐hoseny et al. 2020).

#### Interleukin-6

Elevated levels of Interleukin-6 (IL-6) are associated with inflammatory responses in testicular tissues, contributing to the overall inflammatory response. Exposure to various pesticides, such as paraquat and chlorpyrifos, leads to elevated IL-6 levels in testicular tissues. This increase is associated with the activation of inflammatory pathways that contribute to tissue damage and dysfunction***.*** Elevated IL-6 levels due to pesticide exposure can disrupt normal spermatogenesis. Chronic inflammation mediated by IL-6 may impair the function of Sertoli cells and germ cells, leading to reduced sperm quality and quantity. This is particularly concerning as sustained high levels of IL-6 can contribute to apoptosis of germ cells and affect overall male fertility (Gangemi et al. 2016)*.*

In interaction with other cytokines, IL-6 often interacts with other inflammatory cytokines such as TNF-α and IL-1β. The combined effects of these cytokines create a pro-inflammatory environment that may promote chronic inflammation and reproductive toxicity (Santos et al. 2023). This immunotoxicity can lead to a higher risk of reproductive disorders and long-term health consequences (Lopes-Ferreira et al. 2023)*.* Furthermore, histological studies have shown that increased IL-6 levels correlate with significant damage to testicular architecture, including degeneration of seminiferous tubules and loss of Leydig cells, which are essential for testosterone production and overall reproductive function (Guazzone et al. 2009; Hassan et al. 2021).

IL-6 inhibits the degradation of integral membrane proteins that are essential for maintaining the integrity of the BTB, such as Occludin, JAM-A, and N-Cadherin. This inhibition leads to an accumulation of these proteins on Sertoli cell surfaces, which alters their normal localization and function. It also activates the ERK-MAPK signaling pathway in Sertoli cells, the pathways that regulates the proliferation of immature Sertoli cells but also determines the appropriate number of Sertoli cells in the testes at puberty. This activation is crucial for regulating tight junction dynamics and permeability, contributing to the disruption of BTB integrity during inflammatory responses (Zhang et al. 2014). Elevated IL-6 levels lead to increased expression of suppressor of cytokine signaling 3 (SOCS3), which inhibits the phosphorylation of STAT3. This reduction in STAT3 activity affects gene expression related to spermatogonial differentiation, further contributing to impaired sperm development (Huang et al. 2016).

#### Interleukin-1 beta

Interleukin-1 beta (IL-1β) is a crucial pro-inflammatory cytokine that plays a significant role in the immune response and inflammation. IL-1β is primarily produced by activated macrophages, monocytes, and dendritic cells (Boraschi 2022)***.*** IL-1β is synthesized as an inactive precursor (pro-IL-1β) and requires cleavage by caspase-1 to become active. This process typically occurs within an inflammasome complex, which is activated by pathogen-associated molecular patterns (PAMPs) or danger-associated molecular patterns (DAMPs) (Lopez-Castejon and Brough 2011).

While acute pesticide poisoning is associated with increased levels of inflammatory cytokines, including IL-1β, chronic exposure may lead to dysregulation and a subsequent decrease in this cytokine. This suggests a complex relationship where initial exposure can trigger an inflammatory response, but prolonged exposure may result in altered cytokine profiles (Kim et al. 2022)***.*** The immunotoxic effects of pesticides can lead to changes in systemic cytokine levels, including a potential suppression of IL-1β production over time. This aligns with findings that chronic exposure to pesticides can compromise immune function and alter cytokine levels (Gangemi et al. 2016). Chlorpyrifos, fipronil, mancozeb, and chlorothalonil are associated with increased levels of IL-1β due to their ability to trigger inflammatory responses and disrupt immune cell functions (Lopes-Ferreira et al. 2023). IL-1β is not produced in normal testis, but in infection and inflammation, it can contribute to testicular tissue injury. Intratesticular IL-1β injection in adult rats induces vascular inflammation and a potent inflammatory response (Bergh and Söder 1990). High intratesticular IL-1 and NO levels in rats with varicocele may cause severe spermatogenesis alterations (Amjad et al. 2006).

#### Monocyte chemoattractant protein-1

Monocyte chemoattractant protein-1 (MCP-1) plays a significant role in testicular injury induced by pesticides and insecticides. This chemokine is involved in recruiting monocytes to sites of inflammation, which can further aggravate testicular injury. Moreover, as exposure to pesticides leads to increased production of pro-inflammatory cytokines, including TNF-α and IL-6, MCP-1 can amplify these inflammatory responses by promoting the secretion of additional cytokines from recruited immune cells, creating a feedback loop that further enhances inflammation and tissue injury (Petricca et al. 2023)***.***

Increased MCP-1 levels correlate with significant histopathological changes in testicular tissues, including degeneration of seminiferous tubules and loss of Leydig cells. These changes can disrupt normal testicular function and hormone production (Hu et al. 2021)***.*** IL-6 levels can influence the expression of MCP-1. For example, an increase in IL-6 often precedes a rise in MCP-1, indicating that IL-6 may play a role in inducing MCP-1 expression during inflammatory responses. This relationship suggests a sequential activation of inflammatory pathways where IL-6 acts as an initial trigger for MCP-1 production (Niwa et al. 2016)***.*** Increased levels of both IL-6 and MCP-1 have been associated with apoptosis of germ cells in the testes. The inflammatory environment created by these cytokines can disrupt normal spermatogenesis, leading to reduced sperm quality and quantity (Aubry et al. 2000) (Fig. 4).

#### Neutrophil-to-lymphocyte ratio (NLR)

An increased NLR is indicative of systemic inflammation and has been used as a marker to assess the severity of testicular injury. The activation of inflammatory pathways due to insecticide exposure can lead to increased production of cytokines that promote neutrophil proliferation while potentially suppressing lymphocyte activity. This imbalance could contribute to an elevated NLR. Prolonged exposure to harmful substances like insecticides may result in chronic inflammation, which is characterized by persistent elevation of neutrophils relative to lymphocytes, further supporting the association between high NLR and testicular toxicity (Yaqub 2019; Lopes-Ferreira et al. 2023). Neutrophils release pro-inflammatory cytokines such as IL-6 and TNF-α upon activation. These cytokines activate signaling pathways such as NF-κB and MAPK in testicular cells (Hedger 2011). Their harmful effects are previously mentioned.

## Clinical evidence on insecticides induced-testicular injury

### Human studies

#### Pyrethroid

The possible risks that pyrethroids pose to humans and the environment are receiving a growing amount of scrutiny. It has been determined that pyrethroids are endocrine disruptors that trigger ailments with male reproduction. Pyrethroids harm male reproductive systems, according to several research findings. DNA damage from pyrethroid pesticide use results in a rise in spermatozoa with malformed heads, which is followed by degeneration and mortality (Bao et al. 2020). Through a number of intricate processes, such as blocking steroid synthesis, influencing the hypothalamic-pituitary–gonadal axis, acting as modulators of estrogenic receptors, and causing oxidative stress, these pesticides have harmful effects on the male reproductive system (Wang et al. 2020). According to specific epidemiological investigations, adult male individuals’ reproductive health may be affected by environmental exposure to pyrethroids, primarily in terms of decreased sperm amount, performance, and impaired sperm DNA (Jurewicz et al. 2015; Radwan et al. 2015). Ingestion of food containing pesticide remnants, inhalation of polluted dust and air, and dermal contact are some of the ways that anyone is exposed to pyrethroid pesticides (Saillenfait et al. 2015).

Unfortunately, human urinary samples and breast milk often include pyrethroids or their metabolites (Ye et al. 2017). Urinary pyrethroid metabolite levels are frequently employed as biomarkers to assess human pyrethroid exposure, and a few epidemiological studies have found correlations between these levels and the reproductive hormones generated by the HPG axis. In Chinese male infertility patients, there was a positive connection between serum LH and urinary 3-phenoxybenzoic acid levels, a pyrethroid metabolite (Han et al. 2008). Serum levels of FSH and LH were observed to positively correlate with urine 3-PBA levels in adult males from the United States. Furthermore, trans-(2, 2-dichlorovinyl)−2, 2-dimethylcyclopropane-1-carboxylic acid (trans-DCCA), another pyrethroid metabolite, was found to negatively correlate with testosterone (Meeker et al. 2009). Pyrethroids are thus thought to have inhibitory effects on testicular steroid production, lowering hormone levels and perhaps increasing pituitary FSH and LH production through the HPG axis’s negative feedback loop (Hu et al. 2013). In contrast, Yoshinaga et al. (2014) found no evidence of a significant correlation between 3-PBA and HPG axis hormones in male university students in Japan. Using the biomarker method to identify pregnant women exposed to pyrethroid pesticides, it was found that the meconium of the fetus and the hair of pregnant women have varying quantities of pyrethroid pesticide residues (Fernández-Cruz et al. 2020), supporting another study revealed a correlation between pyrethroids exposition and decreased birth weight, indicating disruption of intrauterine growth (Saillenfait et al. 2015).

According to a comprehensive and meta-analysis evaluation of clinical trials on the impact of pyrethroids on human semen parameters, Lifeng et al. found no difference, although only normal sperm morphology was affected (Lifeng et al. 2006). Therefore, further prospective investigations with a bigger sample size were necessary, as the effect of pyrethroids on sperm head abnormalities relied on two small studies with an overall 27 subjects patients. Additionally, Bian et al. observed a larger percentage of DNA in the sperm tail (Bian et al. 2004), while Xia et al. showed a greater 18% of disomic and sex chromosome spermatozoa (Xia et al. 2004). These findings indicate a detrimental influence on the DNA integrity of human spermatozoa. Other studies showed that the average sperm motility was 40% lower in exposed males than in fertile unexposed ones (Hu et al. 2020; Campbell et al. 2021).

A recent study investigated the endocrine-disrupting potential of five pyrethroid insecticides (allethrin, phenothrin, deltamethrin, cypermethrin, and lambda-cyhalothrin) using an integrated approach to testing and assessment. The study incorporates enzyme-linked immunosorbent assays to measure 17β-estradiol and testosterone levels, along with receptor transactivation assays to assess estrogen receptor and androgen receptor interactions using human H295R adreno-carcinoma cell line for the steroidogenesis genes assay that encode for all the key enzymes for steroidogenesis. All tested pyrethroids substantially reduced T levels and can act as estrogenic agonists and androgenic antagonists. Meanwhile, some compounds showed interesting dual action, where they could activate estrogen receptors but decrease 17β-estradiol levels (Ortiz et al. 2025).

#### Organophosphates (OP)

Organophosphates are common household and agricultural insecticides; nonetheless, they have been connected to low sperm quality and endocrine disruption. Numerous studies have shown that OP insecticides negatively impact the quality of sperm and male reproductive hormones. However, the majority of this research uses animal models, and there is little data on humans to back up this assertion (Mali et al. 2023).

Padungtod et al. found that Chinese pesticide factory workers’ exposure to OP pesticides decreased their sperm concentration and motility (Padungtod et al. 2000). However, Hossain et al., demonstrated that OP pesticide exposure considerably decreased sperm count, motility, and viability (Hossain et al. 2010), while Recio-Vega et al. noted that exposure to OP pesticides significantly decreased ejaculate volume and sperm count but not motility and viability (Recio‐Vega et al. 2008). Interestingly, circulating testosterone, LH, and FSH were unaffected in a cross-sectional investigation among Venezuelan farmworkers showed that exposure to OP pesticides was inversely connected with sperm count, morphology, and viability (Miranda-Contreras et al. 2013). Surprisingly, Ghafouri Khosrowshahi et al. found that OP pesticides dramatically decreased sperm motility and count while increasing serum testosterone. Sperm morphology, ejaculate volume, and semen pH were unaffected (Ghafouri-Khosrowshahi et al. 2019). This is comparable to the results of Kamijima et al. who found that users of OP pesticides had significantly higher serum testosterone levels (Kamijima et al. 2004). Recently, Giulioni’s systematic and meta-analysis review of the effects of OPs on human semen parameters revealed a significant decrease in a number of semen variables, including ejaculate volume, sperm number, and concentration. This finding confirmed the detrimental impact of OPs on human spermatogenesis (Giulioni et al. 2021).

As the underlying mechanisms become clear, OPs change the blood-testis barrier’s physiological function by forming covalent connections with the occluded zone 2 (Ortega-Olvera et al. 2018) and generating reactive oxygen species (Urióstegui-Acosta et al. 2020). An indirect indication of this process was the elevated mean seminal pH value in exposed men. Furthermore, in biochemical and seminal analysis, Miranda-Contreras et al. (2013) and Yucra et al. (2006) reported a greater amount of leucocytes. Other mechanisms involve NRF2 and OGG1, which are two examples of antioxidant and DNA repair gene promoters whose expression is changed by OPs once they enter the nucleus (Hernandez-Cortes et al. 2018). Moreover, OPs weaken DNA integrity and chromatin condensation (Sarabia et al. 2009). According to other studies, exposed men had a greater DNA fragmentation level (Sánchez-Peña et al. 2004; Miranda-Contreras et al. 2013). Additionally, there was a significant decrease in meiotic figures, elongating spermatids, a lack of normal cell-to-cell communication, epithelial cells sloughing, and associated cease of spermatogenesis within the seminiferous tubules (Narayana et al. 2006; Joshi et al. 2007). Furthermore, the polymorphism of Paraoxonase gene, an enzyme that is crucial against anticholinesterase toxicity, was found to be the primary factor in agricultural workers’ vulnerability to chronic OP poisoning (Pérez-Herrera et al. 2008).

Recently, 766 male individuals of reproductive age participated in a different systematic review and meta-analysis on the effect of OPs on human male fertility. The study examined the effects of exposure to one or more OP pesticides for a minimum of six months, whether from residential exposure or occupational environment. The ejaculate volume, seminal fluid volume, sperm multiple anomaly index, and leukocyte levels of the OP-exposed subjects did not change significantly from the control group. Furthermore, exposure to OP insecticides had no discernible effect on serum levels of testosterone, FSH, or LH. However, compared to the unexposed patients, OP pesticide-exposed subjects showed a substantial decrease in sperm count, concentration, motility, and normal sperm morphology, potentially through a mechanism independent of testosterone (Hamed et al. 2023).

Another recent randomized trial investigated N-acetylcysteine as an antioxidant for acute OP poisoning in 56 patients. N-Acetylcysteine significantly increased serum catalase and glutathione peroxidase levels compared to standard treatment alone, but did not significantly affect atropine dosage, treatment duration, ventilation needs, or hospital stay. Mortality was slightly lower in the N-acetylcysteine group, but the difference was not statistically significant. The study concludes that N-acetylcysteine improves antioxidant enzyme levels but does not significantly impact clinically relevant outcomes in acute OP poisoning (Mohammed et al. 2025).

To evaluate the potential human exposure to OPs, A recent study investigated chlorinated organophosphate esters in car and road dust from Basrah, Iraq, finding higher concentrations in car dust and urban areas. Tris (1,3-dichloroisopropyl) phosphate and Tris(2-chloroisopropyl) phosphate were the most abundant compounds. Estimated daily intakes revealed higher exposure for toddlers and taxi drivers, with car dust ingestion significantly exceeding road dust. The findings highlight potential health risks of chlorinated organophosphate esters, which are potentially mutagenic, carcinogenic, neurotoxic and can accumulate in organs such as the liver and testes, inducing tumors (Al-Omran et al. 2025).

#### Neonicotinoids insecticides (NNIs)

For a range of urban and agricultural applications, NNIs, a recently developed family of insecticides, have swiftly risen to the top of the global usage rankings (Myers and Hill 2014; Hladik et al. 2018). Because of their supposed low risk to the environment and non-target creatures, NNIs were once thought to be perfect substitutes for certain insecticides, such as carbamates and organophosphates (Anjos et al. 2021). Few studies, nevertheless, have described the possible harmful health consequences associated with NNIs exposure in humans (Thompson et al. 2020). However, depending on the exposure route and duration, NNIs can have harmful effects on non-target organisms that are either acute or subacute (Bommuraj et al. 2021; Saka and Tada 2021). Due to their extensive use in the environment, NNIs pose serious health risks to both people and ecosystems, upsetting reproductive processes, disrupting ecological equilibrium, and impairing the development of human neural systems (Zuščíková et al. 2023). Furthermore, it has been established that acetamiprid harms the reproductive system in a number of species (Arıcan et al. 2020).

Studies on animals have shown that exposure to NNIs may impair male reproductive function; however, there is a lack of information regarding the presence of NNIs and their particular metabolites in human seminal plasma. A clinical investigation assessed the levels of NNIs and some of its metabolites in seminal plasma samples taken from 191 men who attended a reproductive clinic in North China between 2018 and 2019 to investigate the possible impacts of NNI exposure on the quality of male semen. Desmethyl-acetamiprid, imidacloprid-olefin, and desmethylclothianidin were commonly found in the samples. Reduced progressive sperm motility and semen quality were linked to these metabolite concentrations. To verify these results, more human research is necessary, and the underlying mechanisms require being addressed (Wang et al. 2022).

Urinary NNI concentrations in Chinese teenagers and their correlation with pubertal growth in both males and females were investigated in another cross-sectional study. High detection rates for all NNIs ranged from 72.0 to 100.0% when urinary creatinine was used to adjust concentrations. Early axillary hair development in girls and delayed genital development in boys were linked to higher thiacloprid concentrations. However, no correlations between other urinary NNIs and other puberty markers were discovered. These results imply that exposure to neonicotinoids may have an impact on adolescent pubertal development, which in turn may have a consequence on adult male reproductive efficiency (Yue et al. 2022).

However, the relationship between insecticides and male fertility remains inadequately explored due to a significant lack of comprehensive clinical studies. While existing research suggests that exposure to these chemicals can adversely affect sperm quality and hormone levels, most studies are limited in scale and scope, often focusing on specific populations rather than broader demographics. Notably, many findings indicate a correlation between insecticide exposure and reduced sperm concentrations, yet large-scale epidemiological studies that definitively establish causation are scarce. Furthermore, the methodologies used in current studies frequently present challenges in interpretation, leading to calls for more rigorous research to clarify these associations. Overall, the current body of evidence underscores an urgent need for extensive human studies to accurately assess the real impact of these substances on male reproductive health as collectively displayed in Table 1.

**Table 1:** Human studies involving the effects of various insecticides on testicular function

Human studies						
Agent	Study design	Exposure method	Study population	Duration	Effect	Ref
A. Pyrethroid						
	Cross-sectional study	General	University students	–	No significant association with serum hormone levels	(Yoshinaga et al. 2014)
	Prenatal exposure	Environmental	Placenta and Meconium	–	Maternal and fetal samples contained varying quantities of pyrethroid pesticide	(Fernández-Cruz et al. 2020)
Fenvalerate	Occupational exposure	Air	Workers	–	Compromised Sperm parameters	(Lifeng et al. 2006)
Fenvalerate	Occupational exposure	Air	Workers	–	Sperm DNA damage	(Bian et al. 2004)
Fenvalerate	Occupational exposure	Air	Workers	Plant workers for 6 consecutive months prior to sample collection	Morphologic abnormality and genotoxic defects of spermatozoa	(Xia et al. 2004)
	Cross-sectional study	Environmental	Prospective Fathers	–	Higher 3-phenoxy benzoic acid levels associated with poor semen quality	(Hu et al. 2020)
Human studies						
Agent	Study design	Exposure method	Study population	Duration	Effect	Ref
B. Organophosphates						
Ethyl parathion and Methamidophos	Occupational exposure	–	Workers	Plant workers for 3 consecutive months before sample collection	Decreased sperm concentration and motility	(Padungtod et al. 2000)
Paraquat or Malathion	Cross-sectional study	Occupational	Farmers	–	Decreased sperm count, motility, and viability	(Hossain et al. 2010)
	Longitudinal follow-up study	Occupational	Agricultural Workers	Three main periods of the agricultural cycle	Decreased ejaculate volume and sperm count	(Recio‐Vega et al. 2008)
	Cross-sectional study	Occupational	Agricultural Workers	–	Normal hormonal profile Compromised sperm count, morphology, and viability	(Miranda-Contreras et al. 2013)
	Comparative study	Environmental	Rural farmers and urban men	–	Decreased sperm motility and count	(Ghafouri-Khosrowshahi et al. 2019)
	Comparative study	Occupational	Insecticide sprayers	Summer and the following winter	Increased serum testosterone levels	(Kamijima et al. 2004)
Human studies						
Agent	Study design	Exposure method	Study population	Duration	Effect	Ref
C. Neonicotinoids insecticides						
	Cross-sectional study	Environmental	Men visiting fertility clinics	–	Reduced sperm motility and semen quality associated with metabolite concentrations	(Wang et al. 2022)
	Cross-sectional study	Environmental	Chinese adolescents	–	Early axillary hair development in girls and delayed genital development in boys	(Yue et al. 2022)

### Animal studies

Recently, interest has grown in investigating the antifertility effects of insecticides, particularly their impact on male fertility. Most studies have found a positive correlation between insecticide exposure and changes in semen parameters (Kamijima et al. 2004). The testicular toxicity of insecticides is a significant side effect due to its potential impact on infertility, even though no cases of infertility related to insecticide toxicity have been reported. Animal studies investigating the effects of insecticides on male fertility reveal a complex interplay of mechanisms, including the roles of ROS and inflammation, characterized by damage of sperm cells and impairment of motility and morphology. Additionally, inflammation induced by pesticide exposure can disrupt hormonal balance, affecting testosterone levels and leading to altered reproductive function (Uwamahoro et al. 2024).

Accordingly, numerous animal studies have been carried out to assess the exact mechanisms behind the cellular and molecular damage to the gonads during spermatogenesis after exposure to insecticides (Latchoumycandane et al. 2002). Given the clear cytotoxicity of these insecticides, studying cellular proliferation using rats as an animal model became essential to investigate the potential mechanisms and dosage related to drug-induced cytotoxicity (Cupp et al. 2003). Moreover, ROS primarily consists of superoxide anions, hydrogen peroxide, and singlet oxygen (Kovacic and Somanathan 2008). These oxygen-derived species can attack DNA, leading to a unique pattern of DNA damage. ROS plays a crucial role in the pathogenesis of the male reproductive system, as the presence of polyunsaturated fatty acids in the testes makes this organ particularly vulnerable to oxidative damage (Turner and Lysiak 2008; Mathur and D’cruz 2011).

Many studies have shown that OP-induced ROS leads to damage in reproductive tissues and plays a significant role in testicular pathophysiology, likely by inhibiting various enzymes involved in DNA synthesis and disrupting the polyunsaturated fatty acids in membranes (Kapoor et al. 2011). This results in reduced activity of GSH, GSH peroxidase, and antioxidant vitamins (Uzun et al. 2009; El-Gendy et al. 2010). Additionally, several studies have reported the harmful effects of environmental toxicants, such as the insecticide imidacloprid (IMI), on the reproductive systems of both humans and experimental animals (Pflieger-Bruss et al. 2004).

The reduction in testosterone levels caused by IMI exposure suggests that IMI may directly inhibit testosterone production in Leydig cells. This disruption likely stems from interference in the biosynthesis of testosterone, possibly due to decreased secretion of LH by the pituitary, and a reduction in the transcriptional activity of genes responsible for steroidogenic enzymes (Bal et al. 2012a).Therefore, the negative impact of insecticides on male rat reproduction seems to be a result of oxidative stress induced in the testes. Apoptosis occurs extensively in the testes, with up to 75% of germ cells undergoing spontaneous apoptosis, disrupting normal spermatogenesis (Karabay and Oguz 2005). Similarly, the increased apoptotic index observed in testicular tissue in the current study was strongly associated with reduced sperm count and heightened sperm abnormalities.

Studies involving rats have demonstrated that certain insecticides can reduce Leydig cell size and testosterone production while increasing luteinizing hormone levels, indicating a disruption in the hypothalamic-pituitary–gonadal axis. For instance, a recent study investigated the protective effects of vitamin E and Coenzyme Q10 against acetamiprid-induced male reproductive toxicity in rats. Acetamiprid administration significantly decreased body and testicular weights, sperm count/motility, testosterone, and antioxidant levels, while increasing dead/abnormal sperm and oxidative stress markers. Vit E or CoQ10 alone improved some acetamiprid-induced effects, but not testosterone or weights. Combined Vit E and CoQ10 were most effective in improving body/testicular weights, sperm quality, hormone levels, and reducing apoptosis, suggesting a synergistic protective effect against acetamiprid toxicity (Abd El Rahman et al. 2025). An earlier study has investigated the toxic effects of sub-acute acetamiprid exposure on the epididymis and seminal vesicles of adult rats. Rats were exposed to 1/5 or 1/20 of the LD50 of acetamiprid for 4 weeks. Acetamiprid exposure significantly increased tissue levels of MDA (a marker of oxidative stress) and decreased tissue levels of TAC (a marker of antioxidant capacity). Histopathological studies revealed acetamiprid-induced tissue damage in both organs, with more severe damage observed in the 1/5 LD50 group. The study concludes that sub-acute acetamiprid exposure causes significant biochemical and histopathological damage to the epididymis and seminal vesicles of adult rats, with greater damage at higher doses (Mesallam et al. 2025).

Earlier animal research had also examined the toxicity of the neonicotinoid insecticide acetamiprid on the male reproductive system of rats. The rats were orally administered low, medium, or high doses of acetamiprid for 90 days. The results showed that acetamiprid decreased sperm concentration and testosterone levels in a dose-dependent manner. The study also found that acetamiprid disrupted hormone levels (GnRH, FSH, LH), caused oxidative stress in the testes, and induced apoptosis with histological testicular damage. The study concludes that acetamiprid is toxic to the male reproductive system at higher doses, potentially due to oxidative stress, hormonal imbalances, and apoptosis (Arıcan et al. 2020).

Another recent study investigated the reproductive toxicity of Coragen, a pesticide containing chlorantraniliprole, in male rats. Rats exposed to low and high doses of Coragen for 8 weeks showed dose-dependent disruptions in reproductive hormones, steroidogenic enzymes, and oxidant-antioxidant balance. Coragen exposure caused testicular tissue damage, reduced sperm motility and viability, increased sperm abnormalities, induced apoptosis through caspase-3 and Bax activation, and caused DNA damage. The study concludes that Coragen’s reproductive toxicity is likely due to oxidative stress, hormonal imbalances, apoptosis, and DNA damage, potentially leading to infertility and impaired reproductive function (Mahmoud et al. 2025).

Earlier investigation showed the effects of co-exposure to the fungicide iprodione and the insecticide chlorpyrifos on the development of male rat reproductive organs. The research found that both iprodione and chlorpyrifos individually disrupt reproductive development and function, leading to issues like reduced testicular weight, altered sperm quality, hormonal imbalances, and testicular damage. Importantly, the study revealed that co-exposure to iprodione and chlorpyrifos caused significantly more severe damage than exposure to either pesticide alone, suggesting an additive toxic effect that poses a greater risk to male fertility and highlighting the need to avoid concurrent exposure (Hassan et al. 2021).

The effects of the ubiquitous organophosphate pesticide malathion on the growth of the rat’s epididymis were also examined in rats. Low dosages of malathion were administered to male rats between postnatal days 25 and 65. The findings demonstrated that malathion exposure hampered epididymal growth. In particular, the epididymis experienced tissue remodeling and an increase in inflammatory cell migration because of the higher dose. Meanwhile, the lesser dose changed the levels of immune cytokines and reduced sperm motility. However, the epididymal shape was impacted by both dosages. The study concludes that postnatal epididymal development can be adversely affected by even low-dose exposure to malathion and that a novel discovery associated with malathion’s effects is altered immune responses in the epididymis (Erthal et al. 2020).

In previous investigation, male rats were used to test the pesticide sulfoxaflor for reproductive harm. For four weeks, rats were given different dosages of sulfoxaflor (25, 100, and 500 mg/kg). According to the findings, sulfoxaflor exposure raised sperm levels of FSH, LH, and MDA. It also causes sperm to become less viable, and more aberrant and damages their DNA. According to histopathological investigation, significant damage to the seminal vesicles, the testes, and the epididymis was observed. Based on hormonal alterations, sperm damage, oxidative stress, and tissue damage, the study suggests that sulfoxaflor exposure causes reproductive harm in male rats (Mohamed et al. 2022).

The harmful effects of imidacloprid, a neonicotinoid insecticide, were examined on the reproductive system of neonatal male rats. From postnatal day 1 to day 26, rats received varying dosages of imidacloprid subcutaneously. According to the study, imidacloprid caused significant changes in body weight gain at the highest dose, increased HDL, decreased cholesterol and triglycerides, decreased antioxidant enzymes and increased ROS. In addition, testicular and epididymal histopathological changes were illustrated as reduced spermatogenesis, altered seminiferous tubules, and plasma testosterone levels. Accordingly, exposure to imidacloprid during the neonatal stage can interfere with testicular and epididymal development, affecting spermatogenesis and testosterone production (Sardar et al. 2023).

Another study examined how rutin protects male rats from the reproductive damage caused by the pesticide hexachlorobenzene. For 28 days, rats were given either hexachlorobenzene alone, RUT alone, or a mix of the two. According to the study, hexachlorobenzene adversely affected lipid profiles, disrupted hormone levels (increased FSH and LH, decreased testosterone), and changed apoptosis-related markers (increased Bax, Caspases-3 and −9, decreased Bcl-2). In addition, increased oxidative stress, decreased antioxidant enzyme activity and glutathione levels, and affected sperm quality (reduced count, motility, viability, and increased abnormalities) were also recorded. It also caused histological abnormalities in the testes. Rutin therapy, however, dramatically reduced these negative effects brought on by hexachlorobenzene. The study concluded that rutin protects male rats against the harmful effects of hexachlorobenzene on reproduction (Tijani et al. 2024).

On the other hand, studies involving mice have demonstrated that curcumin exerted a possible ability to shield male mice’s testicles against malathion-induced damage. For four weeks, mice were given either curcumin, malathion, or a combination of the two. According to the findings, exposure to malathion raised oxidative stress, decreased antioxidant enzyme activity, decreased testosterone and LH levels, and damaged the testes histologically. On the other hand, co-administration of curcumin and malathion enhanced spermatogenesis, decreased oxidative stress, and raised testosterone and antioxidant enzyme levels. According to the study’s findings, curcumin’s antioxidant qualities may help reduce the harm that malathion causes to the testicles (Ali and Ibrahim 2018).

Another study examined how oxidative stress contributes to the reproductive damage caused by malathion in male prepubertal mice. Malathion was administered to mice for 30 days. Malathion exposure was found to cause histopathological changes in the testes, alter semen parameters, and induce oxidative stress in the testis and epididymis. Crucially, malathion therapy also reduced the mRNA expression of GPx-4/GPx-5 in the testes and epididymis, and COX isoenzymes in the cauda epididymis. The study concluded that the observed dysregulation of reproductive function in prepubertal male mice is partly caused by the pro-oxidant qualities of malathion (Selmi et al. 2015).

Additionally, hazardous organophosphorus pesticide methyl parathion was found to affect the reproductive health of male mice, with particular attention to sperm quality, DNA integrity, and blood-testis barrier integrity. As demonstrated by testicular shrinkage, a previous study discovered that exposure to parathion resulted in dose-dependent reductions in sperm quality, elevated lipid peroxidation and DNA damage, and disturbed spermatogenesis. Interestingly, parathion weakened the blood-testis barrier integrity, which let a tracer molecule through. These harmful effects were avoided by co-administration of the antioxidant α-tocopherol, demonstrating the importance of oxidative stress. Parathion caused the total proteins in testicular tissue to oxidize, however, it had no effect on the location of blood-testis barrier integrity tight junction proteins. The study comes to the conclusion that parathion damages sperm quality by interfering with the blood-testis barrier integrity through an oxidation-mediated mechanism (Urióstegui-Acosta et al. 2020) as illustrated in Table 2.

**Table 2:** Animal studies involving the effects of various insecticides on testicular function

Animal studies					
Agent	Dose	Duration	Treatment/protection	Effect	Ref
Chlorpyrifos	5.4 mg/kg	28 days	Catechin (20 mg/kg), quercetin (20 mg/kg)	Chlorpyrifos alone increased oxidative stress, and catechin and quercetin decreased oxidative stress	(Kalender et al. 2012)
Iprodione (IPR) and chlorpyrifos (CPF)	200 mg/kg IPR orally and 7.45 mg/kg CPF orally	From postnatal day 23 to 60		Additive effects than individual exposure, reduced testicular weight, altered sperm quality, hormonal imbalances, and testicular damage	(Hassan et al. 2021)
Imidacloprid	0.5, 2, and 8 mg/kg orally	90 days		Increased lipid peroxidation, fatty acid concentrations, and higher rates of abnormal sperm as well as DNA fragmentation	(Bal et al. 2012a)
Imidacloprid	1, 5 and 10 mg/kg) subcutaneously	From postnatal day 1 to 27		Sub-chronic exposure in neonatal male rats induces histopathological changes in reproductive tissues and damages normal testicular functions	(Sardar et al. 2023)
Acetamiprid	12.5 mg/kg, 25 mg/kg, and 35 mg/kg	90 days		Sperm concentration and plasma testosterone levels decreased in a dose-dependent manner	(Arıcan et al. 2020)
Acetamiprid	10 mg/kg/day, 40 mg/kg/day orally	4 weeks		Sub-acute exposure caused significant biochemical and histopathological damage to epididymis and seminal vesicles	(Mesallam et al. 2025)
Acetamiprid	25 mg/kg/day orally	60 days	Vitamin E (100 IU/kg/day) and/or Coenzyme Q10 (75 mg/kg/day)	Acetamiprid significantly decreased testicular weights, sperm count/motility, testosterone, and antioxidant levels, while increasing dead/abnormal sperm Combined Vit E and CoQ10 protected against acetamiprid-induced damage	(Abd El Rahman et al. 2025)
Chlorantraniliprole (Coragen)	500 mg/kg, and 1000 mg/kg	8 weeks		Oxidative stress, hormonal disturbance, apoptosis, and damage to testicular DNA	(Mahmoud et al. 2025)
Malathion	10 mg/kg, 50 mg/kg	40 days		impaired postnatal epididymal development	(Erthal et al. 2020)
Malathion	27 mg/kg	4 weeks	Curcumin (200 mg/kg/day orally)	Malathion decreased testosterone and LH levels, and damaged the testes histologically, Curcumin reversed such effects and increased spermatogenesis	(Ali and Ibrahim 2018)
Malathion	200 mg/kg	30 days		Histopathological changes in the testes, altered semen parameters, and induced oxidative stress in the testis and epididymis	(Selmi et al. 2015)
Sulfoxaflor	25, 100, and 500 mg/kg	4 weeks		Significant increase in FSH, LH, MDA, and GPx levels, increased dead and abnormal sperms. Testicular degeneration with coagulative necrosis	(Mohamed et al. 2022)
Hexachlorobenzene	16 mg/kg	28 days	Rutin (100 mg/kg)	Increased oxidative stress, affected sperm quality, and caused histological abnormalities in the testes. Rutin prevented such effects	(Tijani et al. 2024)
Methyl parathion	3–12 mg/kg/day, i.p	5 days	α-tocopherol (50 mg/kg/day for 5 days oral	testicular shrinkage, dose-dependent reductions in sperm quality, elevated lipid peroxidation and DNA damage, and disturbed spermatogenesis α-tocopherol protects against blood-testis barrier destruction	(Urióstegui-Acosta et al. 2020)

## Natural products and their impact on testicular health

Natural products are gaining interest for their antioxidant capabilities, which may mitigate oxidative stress in the body (Elgindi et al. 2016; Aly et al. 2024; Cusumano et al. 2024; Goher et al. 2024; Zengin et al. 2024). These antioxidant properties play an important role in alleviating pesticide toxicity and maintaining testicular health. In the following part, we will discuss different natural products that have been recently investigated for their possible advantages for testicular health in relation to insecticide toxicity (Table 3).

**Table 3:** Summary of natural products with protective effects against insecticide-induced testicular toxicity

Natural product	Dose of natural product	Insecticide name	Class of the pesticide	Dose of pesticide	Protective effects of the Natural product	Reference
Curcumin	100 mg/kg	Imidacloprid	Neonicotinoid	45 and 90 mg/kg	↑ SOD, CAT, GSR, GPx ↑ GGT, LDH, SDH **↑** 3β-HSD, 17β-HSD **↑** Testosterone production **↑**Total epididymal sperm count, motility, and viability **↓** Sperm abnormalities	(Lonare et al. 2016)
	200 mg/kg	Malathion	Organophosphorus	27 mg/kg	↓ MDA ↓ Lipid peroxidation ↑ SOD, CAT Normal testicular architecture **↓**Necrosis, inflammation	(Ali and Ibrahim 2018)
Curcumin and quercetin	100 mg/kg each	Cypermethrin Deltamethrin	Pyrethroids	2 mg/kg each	↑ SOD, CAT, GSR, GPx **↑** 3β-HSD, 17β-HSD **↑** Testosterone production **↓** Lipid peroxidation (LPO)	(Sharma et al. 2018)
Quercetin	50 mg/kg	Fenitrothion	Organophosphorus	20 mg/kg	**↑** 3β-HSD, 17β-HSD ↑ SOD, CAT **↑** Testosterone **↑** Sperm count and motility	(Saber et al. 2021)
Catechin and quercetin	20 mg/kg each	Chlorpyrifos	Chlorinated organophosphate	5.4 mg/kg	↓ MDA **↑** GPx, GST Normal testicular architecture	(Kalender et al. 2012)
Rutin	100 mg/kg	Hexachlorobenzene (HCB)	Organochlorine	16 mg/kg	↓ MDA ↑ SOD, CAT, GSR, GPx ↑ GGT, LDH, SDH **↑** 3β-HSD, 17β-HSD **↑** Testosterone production **↑** Bcl-2 ↓Caspase-3 and Caspase-9 Improvement of sperm motility, viability, and count	(Tijani et al. 2024)
Hesperetin	100 mg/kg	Nicosulfuron	Sulfonylurea	25 mg/kg	↓ MDA, ACP, ALP, NO ↑GSH Enhanced sperm shape, motility	(Olayinka et al. 2022)
Hesperidin	100 or 200 mg/kg	Abamectin	Macrocyclic lactone	1 mg/kg	**↓** Lipid peroxidation **↑** Glutathione SOD, CAT, and GPx **↓** NF-κB, IL-1β, TNF-α, and IL-6 **↓** Caspase-3, beclin-1, LC3A, and LC3B **↑** Bcl-2	(Gur et al. 2022)
Naringenin	50 mg/kg	Permethrin	Pyrethroids	70 mg/kg	**↑** Testicular weight **↑** Testosterone levels	(Mostafa et al. 2016)
Thymol	30 mg/kg	Imidacloprid	Neonicotinoid	22.5 mg/kg	↓ MDA ↑ GSH, TAC **↑** Sperm count, motility, and the live/dead ratio Strong expression for proliferating cell nuclear antigen (PCNA)	(Saber et al. 2021)
Caffeic acid	10 mmol/kg	Cyhalothrin	Pyrethroids	668 ppm	↓ MDA ↑ SOD, CAT, GSR **↑** Sperm parameters	(Abdallah et al. 2012)
Resveratrol	20 mg/kg	Atrazine	Triazines	**50** mg/kg	**↓** Caspase-3 **↓** iNOS Normal testicular architecture **↓** Testicular fibrosis	(Hassanin et al. 2024)

Natural compounds such as curcumin have been recognized for their preventive properties against oxidative damage in reproductive organs. Sharma et al. evaluate the protective effects of curcumin and quercetin against cypermethrin (CYP) and deltamethrin (DEL)-induced reproductive system impairments. Curcumin and quercetin substantially alleviated the damage caused by CYP and DEL by improving reproductive organ weights, sperm count, motility, upregulation of pituitary–gonadal hormones viz. testosterone, follicle-stimulating hormone (FSH) and luteinizing hormone (LH), and the activity of steroidogenic enzymes that led to improved spermatogenesis and overall reproductive health.

The combined effect of both antioxidants offered more protection than either alone (Sharma et al. 2018). Another study examined whether quercetin supplementation could reduce fenitrothion-induced testicular damage and oxidative stress in male rats. Quercetin showed up-regulation of steroidogenic genes (3β-HSD, 17β-HSD) and antioxidant genes CAT and SOD. It also elevates sperm count, motility, and testosterone levels (Saber et al. 2016). Another study also reported that compared to chlorpyrifos, catechin, and quercetin reduced MDA and enhanced GPx and GST activities. Histopathological investigations demonstrated that both flavonoids restored normal testicular architecture, protecting against pesticide damage (Kalender et al. 2012). An additional study revealed the protective effects of curcumin on malathion reproductive toxicity. Curcumin markedly diminished lipid peroxidation by decreasing MDA levels and enhanced the activities of antioxidant enzymes such as SOD and CAT. Additionally, it contributed to the restoration of normal testicular architecture and function by alleviating necrosis, abnormalities, and inflammation induced by malathion (Ali and Ibrahim 2018). Lonare et al. explore the protective effects of curcumin against imidacloprid toxicity. The protective effects of curcumin are ascribed to its antioxidant properties, which mitigate oxidative stress by increasing CAT, SOD, GPx, and GST levels, thereby supporting the body’s antioxidant defenses. Curcumin significantly elevated the enzymatic activities of 3β-HSD and 17β-HSD, as well as testosterone concentrations in both testis and plasma. Additionally, it improved total epididymal sperm count, motility, and viability while reducing sperm abnormalities (Lonare et al. 2016).

Another flavonoid, Rutin which is found in certain fruits and vegetables, is well-known for its antioxidant properties (Aly et al. 2023). A recent study revealed that co-administration of rutin at a dosage of 100 mg/kg markedly diminished MDA and enhanced antioxidant enzyme levels as SOD, CAT, GPx, GSR activities, and GSH levels compared to the Hexachlorobenzene (HCB) alone group. It also significantly elevated the levels of testicular enzymes such as gamma-glutamyl transferase (GGT), lactate dehydrogenase (LDH), and sorbitol dehydrogenase (SDH). Moreover, significant improvement in the activity of steroidogenic enzymes activities in rats, 3β-hydroxysteroid dehydrogenase (3β-HSD) and 17β-hydroxysteroid dehydrogenase (17β-HSD) along with enhancing testosterone synthesis. Besides, rutin diminished the expression of pro-apoptotic genes Bax, Caspase-3, and Caspase-9 while enhancing the expression of anti-apoptotic gene Bcl-2, signifying a protective impact against cellular apoptosis. Regarding the sperm parameters rutin treatment significantly (*P* < 0.05) mitigated all sperm abnormalities regarding sperm motility, viability, and count in the co-treated group relative to the HCB group (Tijani et al. 2024).

Another study reported the protective effects of hesperetin, a citrus flavonoid against oxidative stress and reproductive toxicity caused by nicosulfuron. Hesperetin treatment significantly decreased MDA, ACP, ALP, and NO levels improved the activities of antioxidant enzymes reduced glutathione (GSH) compared to the nicosulfuron-only group. Additionally, Incorporating hesperetin into nicosulfuron treatment significantly enhanced sperm shape, motility, and testicular ascorbic acid levels (Olayinka et al. 2022). Another recent study explores the protective effects of hesperidin the flavanone glycoside of hesperetin against reproductive toxicity caused by abamectin, a commonly used pesticide. Hesperidin reduced lipid peroxidation, increased glutathione levels in testicular tissue, and increased SOD, CAT, and GPx enzyme activity. MAPK14 reduced the expression of NF-κB, IL-1β, TNF-α, and IL-6, resulting in an anti-inflammatory effect. Bax decreased caspase-3, beclin-1, LC3A, and LC3B and increased Bcl-2 to suppress apoptosis and autophagy. Also, it positively influenced the expression of proteins involved in the JAK2/STAT3 pathway (Gur et al. 2022). Another flavonoid present in Citrus fruits naringenin was explored for its protective effects against testicular damage caused by permethrin, a synthetic pyrethroid insecticide. The combined administration of naringenin with permethrin demonstrated notable enhancements in testicular weight and testosterone levels, suggesting protective benefits against permethrin-induced toxicity (Mostafa et al. 2016).

Phenolic compounds such as Thymol were investigated for their role in the mitigation of oxidative stress and influencing gene expression related to steroidogenesis and apoptosis in the context of imidacloprid (IMI) exposure. Thymol therapy at a dose of 30 mg/kg.b.w.t. markedly decreased oxidative stress indicators such as Malondialdehyde (MDA) and enhanced antioxidant enzyme activity as glutathione (GSH), and total antioxidant capacity (TAC) levels in rats subjected to imidacloprid exposure. It also favorably impacted the mRNA expression of genes related to steroidogenesis as it exhibited strong expression for proliferating cell nuclear antigen (PCNA) immunoexpression (39.09 ± 1.66) while suppressing pro-apoptotic genes as caspase-3 immunoexpression (11.83 ± 0.34), suggesting a protective effect on testicular function. Moreover, the simultaneous administration of thymol to IMI-treated rats significantly raised (*P* < 0.001) sperm count, motility, and the live/dead ratio, while reducing the proportion of aberrant sperm morphology (Saber et al. 2021). The preventive advantages of thymol are ascribed to its capacity to neutralize free radicals, augment antioxidant defenses, and regulate signaling pathways associated with inflammation and apoptosis (Nagoor Meeran et al. 2017). Caffeic acid supplementation can alleviate the adverse impacts of cyhalothrin pesticides on sperm parameters and testicular oxidative injury in male rats. It markedly enhanced sperm parameters and diminished MDA levels, indicating a preventive role against damage caused by oxidation. The group treated with caffeic acid exhibited increased activities of antioxidant enzymes such as SOD and CAT, as well as elevated testicular glutathione contents, in comparison to the control group subjected to cyhalothrin (Abdallah et al. 2012). A recent study investigates the protective effects of resveratrol against testicular toxicity caused by atrazine, a widely used herbicide. Resveratrol therapy markedly diminished caspase activity, thereby reducing apoptotic cell death also, it revealed a reduction in iNOS and mRNA levels. Histopathological analyses indicated that resveratrol preserved normal testicular architecture regarding seminiferous tubules and decreased fibrosis compared to the atrazine-only group (Hassanin et al. 2024).

**Fig. 5:**
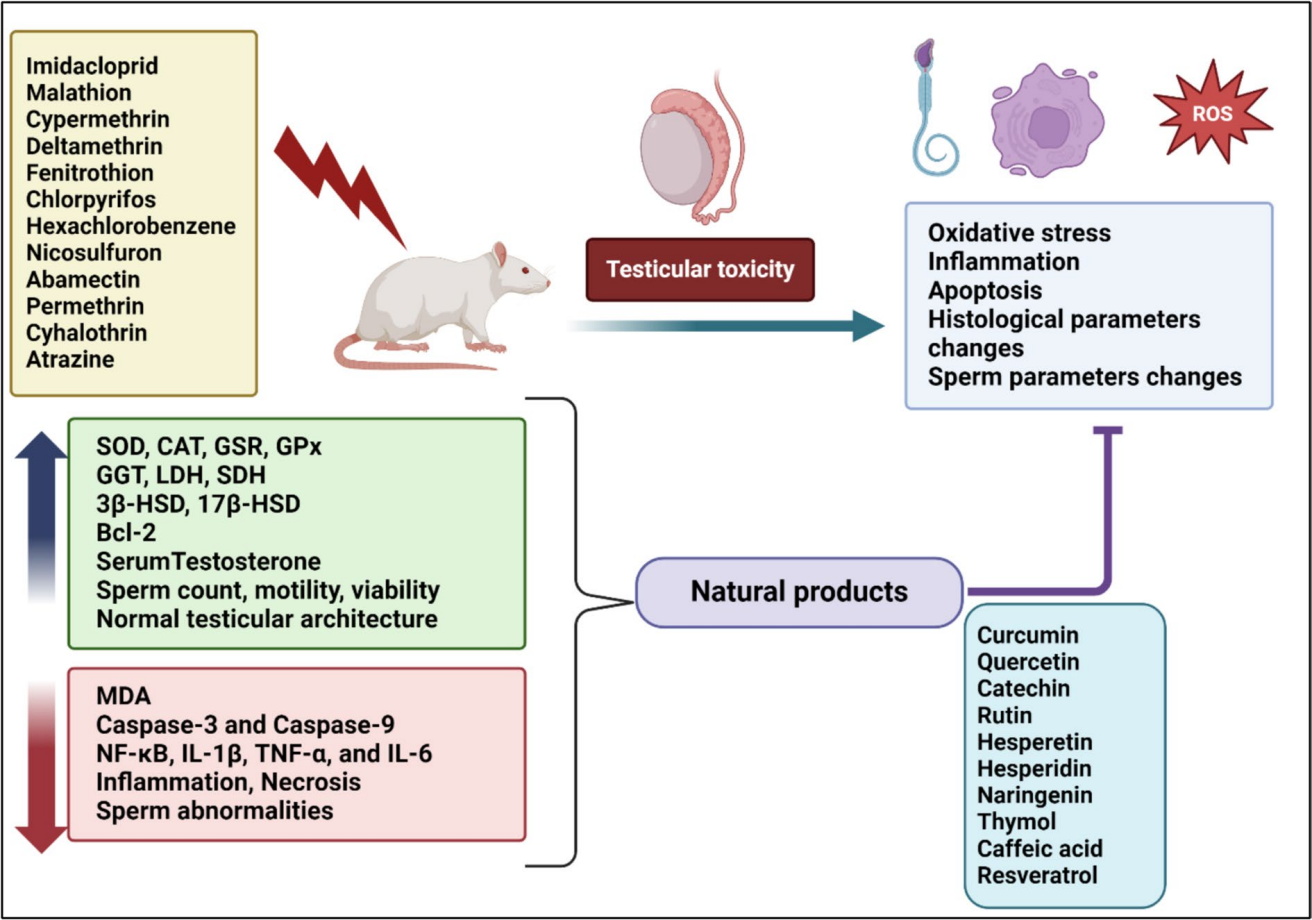
A schematic representation of the putative mechanism of action of natural compounds that may protect against testicular injury caused by pesticides

The previous studies indicate possible therapeutic and protective applications for these phytochemicals in addressing the effects of pesticide toxicity on human fertility. Subsequent investigations could explore long-term benefits under diverse pesticide exposure conditions. Figure 5 illustrates the putative mechanism of action of natural compounds that may protect against testicular damage induced by insecticides.

## Conclusion

Pesticides are currently widely employed in a variety of industries, including healthcare and agriculture, all over the world. This extensive use raises the possibility that both people and animals will be exposed to pesticides, which pose serious health hazards. Numerous medical conditions, including hepatotoxicity, neurotoxicity, and cardiac toxicity, have been linked to pesticide exposure. Furthermore, there is a substantial correlation between pesticides and reproductive problems, namely male infertility. To clarify the underlying mechanisms of pesticide-induced male infertility at a more in-depth molecular level, more investigation is required. Notably, to successfully lessen the detrimental impacts of pesticides on male reproductive health, health education, research projects, and regulatory actions must be given top priority. To promote the judicious use of pesticides and increase knowledge of the health concerns associated with their exposure, it is imperative to comprehend these mechanisms.

## Data Availability

All source data for this work (or generated in this study) are available upon reasonable request.
